# Detecting depression among adolescents in Santiago, Chile: sex differences

**DOI:** 10.1186/1471-244X-13-122

**Published:** 2013-04-23

**Authors:** Ricardo Araya, Jesus Montero-Marin, Sergio Barroilhet, Rosemarie Fritsch, Alan Montgomery

**Affiliations:** 1 School of Social and Community Medicine, University of Bristol, Oakfield House, Bristol, BS8 2PS, UK; 2 Department of Psychiatry, University of Zaragoza, Zaragoza, Spain; 3 Department of Psychology, University of Concepción, Concepción, Chile; 4 Department of Psychiatry, University of Santiago, Santiago, Chile

**Keywords:** Depression, Adolescents, Sex, Beck depression inventory, Screening

## Abstract

**Background:**

Depression among adolescents is common but most cases go undetected. Brief questionnaires offer an opportunity to identify probable cases but properly validated cut-off points are often unavailable, especially in non-western countries. Sex differences in the prevalence of depression become marked in adolescence and this needs to be accounted when establishing cut-off points.

**Method:**

This study involved adolescents attending secondary state schools in Santiago, Chile. We compared the self-reported Beck Depression Inventory-II with a psychiatric interview to ascertain diagnosis. General psychometric features were estimated before establishing the criterion validity of the BDI-II.

**Results:**

The BDI-II showed good psychometric properties with good internal consistency, a clear unidimensional factorial structure, and good capacity to discriminate between cases and non-cases of depression. Optimal cut-off points to establish caseness for depression were much higher for girls than boys. Sex discrepancies were primarily explained by differences in scores among those with depression rather than among those without depression.

**Conclusions:**

It is essential to validate scales with the populations intended to be used with. Sex differences are often ignored when applying cut-off points, leading to substantial misclassification. Early detection of depression is essential if we think that early intervention is a clinically important goal.

## Background

Depression is a common condition affecting people of all ages and races [1], with high prevalence among youngsters in Latin America [2-4]. Early onset depression is of interest because of the need to identify early cases of depression and potentially prevent or reduce consequences later in life [5,6]. Between 20% to 33% of those who meet criteria for the diagnosis of lifetime major depression report that their first episode occurred before the age of 21 [6-9], with a mean age of onset in this group estimated as 15 years [10]. Different studies have shown that depression in adolescence (early onset) affects school performance, increases antisocial behavior, self-harm and suicidal risk; as well as impairing overall functioning [9,11-19].

Notwithstanding the importance of early identification of this disorder, community surveys consistently show that adolescent depression is under-diagnosed and undertreated [20-22]. Screening for depressive symptoms among adolescents may be one way of improving early detection. There are advantages and disadvantages in doing so [23] but identification is a necessary preliminary step if one wishes intervening early [24] with the aim of potentially ameliorating adverse outcomes later in life.

Brief depression self-rating scales can be especially useful for this purpose [25]. The Beck Depression Inventory (BDI) is one of the best known and most widely used self-rating scales to assess the presence and severity of depressive symptoms [26]. The second version of this scale (BDI-II) was created to establish a clearer link with the DSM-IV classification as well as informing on the severity of depressive symptoms. The studies published, mostly for the English version, show good agreement between this questionnaire and the clinical diagnosis of depression [26-28] and good psychometric properties for the scale [26].

The BDI-II when used among adolescents has also shown good psychometric properties [29-39]. However, many of the studies assessing the usefulness of BDI-II with adolescents have been affected by significant methodological limitations. Among these are: small and often only clinical samples, no concomitant assessment with a gold standard and when this is done there are often long delays between the screening and diagnostic interview, and overall poor reporting of methods [24,40]. Needless to say, few studies have been conducted in low and middle income countries where almost 90% of the world’s young population lives.

Among the few studies that have explored BDI-II psychometric properties on adolescent non-clinical samples very few have tested criterion validity. More specifically we were unable to find any studies that had validated the BDI-II against a psychiatric interview (criterion) among adolescents in Latin America. More research is needed on the use of the BDI-II with adolescents from other nationalities and ethnic groups before we can confidently support its use as a screening or case identification tool for youngsters across different cultures.

In Chile, the prevalence of depressive symptoms among adolescents is high compared to other countries [41]. A number of studies with different methodologies have reported prevalence rates ranging from 13% [37] to 44% [42]. A recent study using the BDI-II in a representative urban sample of 700 high-school adolescents found that 33% of these youngsters scored 19 or above on the BDI-II [41]. However, the criterion validity of BDI-II among adolescents has never been studied in Chile and there is no empirical evidence to support the validity of any cut-off points used to define caseness with young populations in that setting or indeed in Latin America.

Sex differences in the prevalence of depression have been extensively reported and they become well established in adolescence. When reaching mid-adolescence there is a shift from similar rates of depression in pre-adolescent boys and girls to approximately twice as many females than males with depression [43] and these differences continue until late in life. There is controversy as to whether or not these are real differences or simply measurement artifacts. Misclassification of questionnaires according to various features has been repeatedly reported [44-46]. The possibility that boys and girls may respond differently to psychiatric questionnaires has been relatively untested even though this may have important repercussion in the estimates obtained when using questionnaires.

This study aims to fill this gap and assess the criterion validity of the BDI-II, determining the best cut-off points for male and female adolescents in Santiago, Chile. Of particular interest is to study possible differences between sexes. In addition this study aims to assess other psychometric properties of the BDI-II.

## Methods

### Sampling and procedures

Fifteen state high schools in Santiago, Chile, participated in this study undertaken in November 2009 and November 2010. Students were being assessed as part of a randomised controlled trial [47], which was concurrently taking place in these schools. The study sample consisted of 592 participants with a mean age 15.5 (SD=0.98), almost half (53.6%) were girls, all of them attending Grade 10th (approximately 10 years of education) in these schools. Two samples were drawn using different methods. The first sample of 250 students was drawn based on their BDI-II scores collected as part of the baseline assessment in five schools in the active arm of the trial. The first 50 students with BDI-II scores between 0 and 6 (lower tertile), the first 100 students whose scores in the middle tertile (7/15), and the first 100 students with high scores (>15) were invited for a clinical interview. For the second sample, all the 352 students in the control arm of the trial who scored high (≥15 for girls and ≥10 for boys) on the BDI-II were invited for clinical interviews. Students answered the BDI-II in the classroom and clinical interviews were performed within 72 hours in a private office in the school for both samples. One of three trained clinicians blinded to the student’s BDI-II status administered this psychiatric interview. In order to improve the blinding of the assessors, interviewers were rotated between schools, so that no-one who participated in the administration of the BDI-II in a particular school also interviewed in the same school.

### Ethics

The study complied and was conducted in accordance with the local Research Governance requirements about ethic concerns, and was carried out in compliance with the Helsinki Declaration. Full ethical approval was obtained from the local Committee (Hospital Clinico Universidad de Chile). At the start of the project a letter was sent to the carers of all eligible young people informing them about the study. The letter therefore informed carers that they could opt out of the assessments if they did not wish their child to complete the questionnaires or the interview. In addition, written consent was obtained before completing the questionnaire or the interview (dual carer/child consent/assent was required).

### Instruments

#### The Beck Depression Inventory-II (BDI-II)

This questionnaire has 21 items asking about depression symptoms experienced over the last two weeks [26]. Answers to each item are on a scale from 0 to 3. For example, ‘I do not feel sad’ (0), ‘I feel sad’ (1), ‘I am sad all the time and I can't snap out of it’ (2), and ‘I am so sad and unhappy that I can't stand it’ (3). The scores to each item are summed to generate a total score with a range between 0 and 63. Cut-off scores are often used to categorize degrees of severity of depression or if a given score matches the presence of a clinical diagnosis. It is highly desirable that cut-off points are established with a population similar to where those cut-off points will be subsequently applied. Traditional cut-off points used to estimate severity in adults are: 10–16 indicating possible mild depression, 17–29 likely moderate depression; and 30–63 probable severe depression [26]. A Spanish translation of the BDI-II showed good psychometric properties when used with US Spanish speaking young populations [48,49]. A Chilean adaptation of the Spanish version of the BDI-II for use with adolescents showed good internal consistency and test-retest correlation coefficients, as well as good concurrent validity with other depression scales and an adequate goodness-of-fit in the confirmatory factor analysis for both uni- and bi-factorial solutions [36]. Several other depression scales were tested in the formative phase but BDI-II performed as good, if not better, than other scales.

#### The Mini International Neuropsychiatric Interview for Children and Adolescents (MINI-KIDS)

The MINI-KIDS [50] is a brief, structured diagnostic interview used to assess the presence of the most common DSM-IV and ICD-10 child and adolescent psychiatric disorders (ages 6 to 16). It follows a similar format as the MINI for adults which was developed as a simpler and briefer psychiatric interview to use for clinical or research purposes [51]. It is reported that the MINI-KIDS generates psychiatric diagnoses for children and adolescents in a third of the time as the K-SADS-PL. It has been translated into Spanish and used extensively in Chile [52,53]. Studies have confirmed good psychometric properties when used among adolescents in different languages with sensitivity of 0.61–1.00 and specificity of 0.73–1.00 for most DSM-IV disorders [50]. It is desirable that interviewers have clinical experience and previous training in the use of this interview.

#### The Revised Child Anxiety and Depression Scale (RCADS)

The RCADS [54] is an adaptation from the Spence Child Anxiety Scale (SCAS) [55] and intends to assess symptoms of DSM-defined anxiety disorders and major depression. The brief version of the RCADS consists of five subscales with five items each one, ranged from 0 (never) to 3 (always), on a 4-point Likert scale [56]. We only included the Spanish version of the generalized anxiety, social phobia, and panic subscales in this study [57]. We excluded the depression and separation anxiety sub-scales because depression was measured with BDI-II and separation anxiety was regarded as less important for this age. Although we are unaware if other researchers have used a similar method we felt that as an approximation to estimating levels of anxiety this is a reasonable approach. We used in the analysis a total score by adding all item scores. The internal consistency of total RCADS scores in this study yielded a value of α=0.84 (males α=0.81; females α=0.84).

### Data analysis

The analysis plan contemplated first to examine the general psychometric properties of the scale in order to determine how best to treat overall scores. Once this is established we will assess the criterion validity of the scale with a view to ascertain the best cut-off points to establish depression, with special emphasis on exploring sex differences.

Firstly, descriptive statistics including means and standard deviations were undertaken and sex differences examined. Subsequently we performed psychometric tests to investigate the performance of BDI-II. Initially we estimated Mardia's coefficients [58] to assess the multivariate normality distribution of the variables. Polychoric correlation is advised for factorial analysis when the distributions of ordinal items are asymmetric or with excess of kurtosis [59]. Thus, a polychoric correlation matrix of BDI-II items was estimated. An unweighted least squares factor analysis (ULS) was the method for factor extraction used in our exploratory factor analysis (EFA) in view of its robustness to failure of normality and heteroscedasticity of the data. We used parallel analysis [60] to identify the number of factors to include in the factorial solution, through replacing the raw data method [61] by optimal implementation based on minimum rank factor analysis [62], generating 500 random correlation matrices. With this analysis, a factor is considered significant if the associated eigen value is bigger than that corresponding to a given percentile, such as the 95th of the distribution of eigen values derived from a random dataset. This method is considered the best available solution to decide the number-of-factors-to-retain for a given scale [63,64]. We tested the goodness of fit of the exploratory model using goodness of fit index (GFI) [65] and root mean square of residuals (RMSR), taking into account Kelley's criterion [66].

Subsequently we performed an invariance analysis according to sex, using confirmatory factor analysis (CFA) and applying generalized least squares (GLS) method. This method is robust and allows estimation of χ^2^ (df), adjusted goodness-of-fit index (AGFI), root mean square error of approximation (RMSEA) (90% CI), standarized root mean square residual (SRMR) and Hoelter_05_ indices. In view that χ^2^ estimations are highly sensitive to sample size we also used χ^2^/df, which indicates a good fit when values are <3 [67,68]. GFI and AGFI refer to explained variance and values ≥0.9 are considered acceptable [65,69]. RMSEA is a measurement of the error of approximation to the population and is considered to be acceptable with values <0.06 [65]. SRMR is the standardized difference between the observed and the predicted covariance, indicating a good fit with values <0.08 [68]. The Hoelter index indicates the sample size required to accept the hypothesis with perfect adjustment and a result of 200 or better indicates a satisfactory fit. In an analysis of multiple groups, it has been suggested that a threshold of 200 times the number of groups is sufficient [70].

We examined the reliability of the scale using congeneric, tau-equivalent, and parallel models, in the total sample and the sample divided by sex. The congeneric model is the least restrictive, and assumes that each individual item measures the same latent variable, with possibly different scales, degrees of precision and magnitude of error. The tau-equivalent model implies that individual items measure the same latent variable, on the same scale, with the same degree of precision, but with possibly different degrees of error. The parallel model is the most restrictive measurement model, and assumes that all items must measure the same latent variable, on the same scale, with the same degree of precision, and with the same amount of error [71]. We finally chose the model that fitted better with the data, applying GLS method, and establishing comparisons between models from the least to the more restrictive, through Δχ^2^. The reliability value was estimated by squaring the implied correlation between the composite latent true variable and the composite observed variable, to arrive at the percentage of the total observed variance that were accounted for by the “true” variable [72]. Item-total correlation coefficients (excluding the same item in the total score), mean inter-item polychoric correlations, and mean item-total correlations (excluding the same item) were also used to assess the internal consistency. Convergent-discriminant validity was assessed comparing the BDI-II with RCADS through Spearman's R coefficient.

Criterion validity was assessed plotting Receiving Operating Characteristics (ROC) curves, comparing the BDI-II with MINI-KIDS for the whole sample, as well as for males and females separately. Of primary interest here was the area under the curve (with 95% CI) as representing the capacity of the BDI-II to discriminate between cases and non-cases according to diagnoses ascertained with MINI-KIDS. We plotted curves for both sexes separately and compared these differences using χ^2^ tests. Sensitivity, as an index of case identification, and specificity, as an index of non-case recognition, were estimated for several cut-off points, in order to ascertain the best trade-off between sensitivity and specificity. Positive and negative predictive values were also estimated, to ascertain the capacity of the questionnaire to detect true and false cases. Finally, we included the Youden Index, which is unaffected by prevalence, and represents the difference between the proportions of true cases and false cases identified by the questionnaire, with a higher the value indicating a better the cut-off point.

Finally we compared the means of the BDI-II and RCADS for cases and non-cases of depression according to the MINI-KIDS in order to explore if sex differences applied to other psychological questionnaires and/or the presence of depression. Given the multiple comparisons in this analysis we used 99% CIs. All analyses were done with SPSS 15.0, Epidat 3.1, Factor 8.02 and Amos 7.

## Results

### Descriptive statistics

Less than 5% of the selected sample needed to be replaced, either because of unwillingness to participate or not attending the day of the interview. Table 1 shows descriptive statistics for BDI-II items and total scores. Mean total scores for boys were significantly lower than for girls [boys=15.33 (8.50) vs. girls=22.78 (10.76); p<0.001)]. Girls had significantly higher mean scores than boys in all items with the exception of ‘pessimism’ (p=0.061), ‘punishment’ (p=0.068), and ‘agitation’ (p=0.529). The largest differences according to sex were found for ‘crying’ [boys=0.56 (1.00) vs. girls=1.56 (1.12); p<0.001]. The skew and kurtosis values showed in general a non-normal distribution of data (data not shown but available from the authors).

**Table 1:** Means and item-total correlations of Beck Depression Inventory (BDI-II) according to sex in a sample of adolescents attending secondary schools in Santiago, Chile

		**Mean (SD)**			**Item-total**		
**BDI items**	**Total**	**Boys**	**Girls**	**p**	**Total**	**Boys**	**Girls**
1. Sadness	0.72 (0.89)	0.48 (0.81)	0.94 (0.90)	<0.001	0.59	0.55	0.56
2. Pessimism	0.68 (0.91)	0.60 (0.85)	0.75 (0.96)	0.061	0.49	0.53	0.47
3. Past failure	0.74 (0.82)	0.59 (0.77)	0.87 (0.84)	<0.001	0.52	0.48	0.51
4. Loss of pleasure	0.79 (0.76)	0.68 (0.75)	0.89 (0.76)	<0.001	0.49	0.46	0.49
5. Guilty	0.85 (0.74)	0.72 (0.69)	0.96 (0.77)	<0.001	0.52	0.44	0.55
6. Punishment	0.91 (1.09)	0.79 (0.97)	1.02 (1.18)	0.068	0.38	0.33	0.39
7. Self-dislike	0.97 (0.95)	0.77 (0.88)	1.14 (0.98)	<0.001	0.52	0.49	0.50
8. Self-criticalness	1.04 (0.94)	0.76 (0.84)	1.29 (0.95)	<0.001	0.55	0.42	0.56
9. Suicidal ideas	0.68 (0.86)	0.46 (0.72)	0.87 (0.92)	<0.001	0.50	0.46	0.46
10. Crying	1.10 (1.18)	0.56 (1.00)	1.56 (1.12)	<0.001	0.43	0.31	0.37
11. Agitation	0.93 (0.91)	0.89 (0.86)	0.96 (0.94)	0.529	0.34	0.32	0.38
12. Loss of interest	0.88 (0.97)	0.69 (0.85)	1.03 (1.04)	<0.001	0.52	0.37	0.56
13. Indecisiveness	0.88 (0.94)	0.70 (0.85)	1.03 (0.99)	<0.001	0.43	0.37	0.42
14. Worthlessness	0.81 (0.90)	0.62 (0.81)	0.97 (0.95)	<0.001	0.60	0.54	0.61
15. Loss of energy	0.97 (0.87)	0.68 (0.73)	1.21 (0.90)	<0.001	0.56	0.42	0.55
16. Insomnia	1.40 (0.95)	1.28 (0.93)	1.51 (0.96)	0.004	0.36	0.28	0.38
17. Irritability	0.92 (0.92)	0.71 (0.82)	1.11 (0.96)	<0.001	0.49	0.32	0.52
18. Loss of appetite	1.32 (0.99)	1.14 (0.96)	1.48 (1.00)	<0.001	0.41	0.28	0.45
19. Concentration difficulties	1.32 (0.87)	1.13 (0.84)	1.49 (0.85)	<0.001	0.45	0.37	0.45
20. Tiredness or fatigue	0.94 (0.88)	0.74 (0.80)	1.11 (0.90)	<0.001	0.60	0.54	0.60
21. Loss of libido	0.48 (0.88)	0.36 (0.74)	0.43 (0.98)	0.008	0.32	0.31	0.29
**Total**	19.32 (10.45)	15.33 (8.50)	22.78 (10.76)	<0.001			

Total n=592; Boys n=275; Girls n=317.

RCADS mean total score was 22.16 (8.60), with boys showing lower mean scores than girls [boys mean = 19.64 (7.98) vs. girls mean = 24.31 (8.53); p<0.001].

### Factorial validity

The analysis of the Mardia's multivariate asymmetry showed a non-normal multivariate distribution of the data for the total sample (kurtosis coefficient = 555.66; p = <0.001) and boys and girls separately. The polychoric correlation matrices of the BDI-II (Additional file 1) revealed that 46.7% correlation coefficients were ≥ 0.30 (38.1% in boys and 38.1% among girls). The determinant of the matrix was 0.01, KMO test had a value of 0.94, and Bartlett's statistic was 3,672.30 (df = 210; p < 0.001), with similar values for boys and girls. Based on these results an EFA for the total sample and according to sex, was undertaken. The parallel analysis based on minimum rank factor analysis (Table 2) identified a clear one factor structure, with an Eigen value of λ_1_ = 7.10, explaining 33.8% of the variance based on eigenvalues (boys λ_1_ = 6.78, 32.3% of the variance; girls λ_1_ = 6.55, 31.2% of the variance). The goodness of fit statistics was good, for the total sample and sub-samples by sex, with values of GFI of 0.99 and 0.04 for RMSR, in keeping with Kelly's criterion.

**Table 2:** Parallel analysis and percentage of variance explained by each factor of the BDI-II

		**Total**			**Boys**			**Girls**	
**Factor**	**Real data**	**Mean random**	**P**_**95**_**random**	**Real data**	**Mean random**	**P**_**95**_**random**	**Real data**	**Mean random**	**P**_**95**_**random**
1*	39.2	13.0	14.7	37.9	16.7	18.3	38.2	10.7	12.0
**2**	6.1	8.6	9.8	7.3	8.4	9.2	6.7	8.9	9.9
**3**	5.4	7.9	8.8	6.0	7.7	8.3	6.0	8.3	9.0
**4**	5.2	7.5	8.2	5.4	7.2	7.7	5.2	7.8	8.4
**5**	4.6	7.0	7.8	5.3	6.8	7.2	5.0	7.3	7.9
**6**	4.2	6.6	7.2	4.9	6.3	6.7	4.7	6.8	7.3
**7**	4.0	6.2	6.8	4.5	5.9	6.3	4.5	6.4	6.8
**8**	4.0	5.8	6.4	4.2	5.5	5.9	4.2	6.0	6.4
**9**	3.6	5.4	5.9	4.0	5.1	5.5	3.8	5.5	6.0
**10**	3.4	5.0	5.4	3.5	4.8	5.1	3.6	5.1	5.5
**11**	3.2	4.6	5.0	3.3	4.4	4.7	3.2	4.7	5.1
**12**	3.0	4.2	4.7	2.9	4.0	4.3	3.0	4.3	4.7
**13**	2.6	3.8	4.3	2.7	3.6	4.0	2.4	3.8	4.3
**14**	2.5	3.4	4.0	2.1	3.2	3.6	2.2	3.4	3.9
**15**	2.2	2.9	3.7	1.8	2.8	3.3	2.1	3.0	3.5
**16**	1.7	2.5	3.2	1.5	2.4	2.9	1.7	2.5	3.1
**17**	1.7	2.1	2.9	1.0	2.0	2.5	1.4	2.0	2.6
**18**	1.5	1.6	2.4	1.0	1.6	2.1	0.9	1.6	2.2
**19**	1.0	1.2	1.9	0.6	1.1	1.6	0.7	1.1	1.6
**20**	0.7	0.7	1.5	0.3	0.6	1.1	0.5	0.7	1.1
**21**	0.0	0.0	0.0	0.0	0.0	0.0	0.0	0.0	0.0

* Advised number of factors; Real data = percentage of the variance explained in relation to the real data based on minimum rank factor analysis; Mean random = mean percentage of the variance explained by the random samples; P_95_ random = percentage of the variance explained over the 95 percentile of the random samples.

Table 3 shows the unrotated loading matrix as well as the communality values from EFA for the total sample, and the standarized weights and standard errors for the subsamples from CFA. All the items loaded strongly and positively in a single factor. In general, the weight of the items ranged from 0.34 for ‘insomnia’ to 0.70 for ‘sadness’, with important differences between sexes in items such as ‘crying’; ‘insomnia’; ‘loss of appetite’; and ‘loss of libido’. Communality values ranged from 0.12 for ‘insomnia’ to 0.48 for ‘sadness’ and ‘worthlessness’ in the total sample. Standard errors were lower among boys than girls, especially for the items ‘loss of libido’ and ‘crying’.

**Table 3:** Factorial weights for each item of the BDI-II according to sex

	**Total**		**Boys**		**Girls**	
**BDI items**	**w**	**c**^**2**^	**w**	**SE**	**w**	**SE**
1. Sadness	0.70	0.48	0.77	0.03	0.66	0.04
2. Pessimism	0.59	0.35	0.72	0.03	0.60	0.05
3. Past failure	0.61	0.37	0.73	0.03	0.60	0.04
4. Loss of pleasure	0.56	0.32	0.65	0.03	0.56	0.03
5. Guilty	0.59	0.35	0.61	0.02	0.62	0.03
6. Punishment	0.46	0.21	0.50	0.06	0.58	0.07
7. Self-dislike	0.60	0.36	0.65	0.04	0.61	0.05
8. Self-criticalness	0.59	0.35	0.55	0.04	0.60	0.05
9. Suicidal ideas	0.61	0.37	0.67	0.02	0.62	0.04
10. Crying	0.49	0.24	0.58	0.06	0.41	0.08
11. Agitation	0.39	0.16	0.41	0.05	0.45	0.06
12. Loss of interest	0.58	0.34	0.55	0.04	0.66	0.05
13. Indecisiveness	0.51	0.26	0.53	0.04	0.51	0.05
14. Worthlessness	0.69	0.48	0.72	0.03	0.71	0.04
15. Loss of energy	0.61	0.37	0.59	0.03	0.62	0.04
16. Insomnia	0.34	0.12	0.29	0.06	0.38	0.06
17. Irritability	0.56	0.31	0.47	0.04	0.61	0.05
18. Loss of appetite	0.41	0.17	0.33	0.06	0.50	0.06
19. Concentration difficulties	0.45	0.20	0.43	0.05	0.46	0.04
20. Tiredness or fatigue	0.63	0.40	0.66	0.03	0.64	0.04
21. Loss of libido	0.47	0.22	0.59	0.03	0.48	0.06

EFA over total and CFA over boys and girls; w = standarized weights; c^2^ = communalities; SE = standard errors.

### Invariance analysis

Adjusting by sex did not alter our main results (Table 4). Good results were also seen when comparing sexes using models without and with restrictions, such as unconstrained, factorial weights, variances or residuals. An analysis including all restrictions at the same time yielded values of χ^2^/df = 1.55; GFI = 931; AGFI = 0.924; RMSEA = 0.031 (90% CI = 0.026-0.035); SRMR = 0.071 y Hoelter = 426. Not with standing these adjustments, χ^2^ values increased significantly when comparing the model without restrictions with the model with restricted residuals (Δχ^2^=93.13; df=21; p<0.001).

**Table 4:** Analysis of invariance according to sex

**Sample**	**χ**^**2**^	**df**	**χ**^**2**^**/df**	**GFI**	**AGFI**	**RMSEA**	**(90% CI)**	**SRMR**	**Hoelter**
Boys	280.15*	189	1.48	0.976	0.971	0.042	0.031 - 0.052	0.053	218
Girls	320.20*	189	1.69	0.976	0.970	0.047	0.038 - 0.056	0.051	220
**Invariance**									
Unconstrained	526.61*	378	1.39	0.975	0.970	0.026	0.020 - 0.031	0.056	477
Weights	548.35*	398	1.38	0.970	0.965	0.025	0.020 - 0.030	0.067	481
Variances	526.77*	379	1.39	0.975	0.970	0.026	0.020 - 0.031	0.056	478
Residuals	619.73*	399	1.55	0.972	0.967	0.031	0.026 - 0.035	0.060	427
All restrictions	651.03*	420	1.55	0.931	0.924	0.031	0.026 - 0.035	0.071	426

*p<0.001. Assuming model unconstrained to be correct: weights Δχ^2^=21.75 (df=20) p=0.354; Variances Δχ^2^=0.16 (df=1) p=0.687; Residuals Δχ^2^=93.13 (df=21) p<0.001.

### Reliability

Table 5 shows the adjusted reliability models tested. The results fitted best with the congeneric model in all the indices, and the Tau-equivalent showed significant increments in χ^2^ (total sample: Δχ^2^=91.60; df=20; p<0.001; boys: Δχ^2^=45.45; df=20; p=0.001; girls: Δχ^2^=50.39; df=20; p=0.001). Based on the congeneric model, the estimates of reliability obtained for the total sample were 0.90; with 0.86 for boys and 0.90 for girls respectively.

**Table 5:** Reliability analysis according to sex

**Sample / model**	**R**	**χ**^**2**^**(df)**	**χ**^**2**^**/df**	**GFI**	**AGFI**	**RMSEA**	**(90% CI)**	**SRMR**	**Hoelter**
**Total**									
Congeneric	0.90	358.28* (189)	1.90	0.942	0.929	0.039	0.033 - 0.045	0.049	367
Tau-equivalent	0.81	449.87* (209)	2.15	0.928	0.920	0.044	0.039 - 0.050	0.072	321
Parallel	0.86	794.79* (229)	3.47	0.872	0.871	0.065	0.060 - 0.070	0.076	198
**Boys**									
Congeneric	0.86	261.81* 189)	1.39	0.909	0.889	0.037	0.026 - 0.048	0.069	233
Tau-equivalent	0.81	307.26* (209)	1.47	0.893	0.882	0.041	0.031 - 0.051	0.074	218
Parallel	0.85	465.46* (229)	2.03	0.838	0.837	0.061	0.053 - 0.069	0.083	157
**Girls**									
Congeneric	0.90	264.79* 189)	1.40	0.920	0.902	0.036	0.025 - 0.045	0.062	266
Tau-equivalent	0.86	315.18* (209)	1.51	0.905	0.895	0.040	0.031 - 0.049	0.071	245
Parallel	0.88	516.22* (229)	2.25	0.844	0.843	0.063	0.056 - 0.070	0.079	163

*p<0.001. From congeneric, In Total sample, Tau-equivalent Δχ^2^=91.60 (df=20) p<0.001 and Parallel Δχ^2^=344.91 (df=20) p<0.001; in Boys sample, Tau-equivalent Δχ^2^=45.45 (df=20) p=0.001 and Parallel Δχ^2^=158.20 (df=20) p<0.001; in Girls sample, Tau-equivalent Δχ^2^=50.39 (df=20) p=0.001; Parallel Δχ^2^=201.05 (df=20) p<0.001. R=reliability;

The mean inter-item correlation was 0.30 for the total (0.28 for boys and 0.27 for girls). The mean item-total correlation was 0.48 for the whole sample (0.41 for boys and 0.48 for girls). All items were positively correlated to the total score, with coefficients item-total (Table 1) ranging from 0.29 (‘loss of libido’ among girls) to 0.61 (‘worthlessness’ among girls). In general, boys had lower values in all item-total correlations, with the exception of ‘pessimism’, ‘loss of libido' and ‘suicidal ideas’.

### Convergent-discriminant validity

The Spearman correlation coefficient between RCADS and BDI-II was 0.46 (p<0.001), with similar coefficients for boys [R = 0.41 (95% CI=0.30-0.50)] and girls [R = 0.43 (95% CI=0.33-0.52)]. Mean BDI-II scores of non-cases [13.50 (7.58)] and cases [24.22 (10.18)] of major depression according to the MINI-KIDS for the total sample were significantly different (p<0.001). Similarly, mean RCADS scores for non-cases [18.13 (7.24)] and cases [25.32 (8.18)] were also significantly different (p<0.001).

Table 6 displays the mean scores of BDI-II and RCADS of cases and non-cases of major depression for boys and girls. The differences in BDI-II means between cases and non-cases are more marked among girls [depressed-girls (n=204): Mean=26.67 (se0.71) vs. non-depressed-girls (n=103): Mean=14.93 (se0.75); p<0.001], than boys [depressed-boys (n=97): Mean=19.09 (se0.83) vs. non-depressed-boys (n=167): Mean=12.61 (se0.58); p<0.001]. Sex differences in BDI-II scores among cases of depression [depressed-boys vs. depressed-girls; Mean-difference=7.58 (99% CI=4.76-10.41); p<0.001], were much larger than those among non-cases [non-depressed-boys vs. non-depressed-girls; Mean-difference=2.32 (99% CI=−0.12-4.75); p=0.014]. In other words much of the difference in mean BDI-II values between boys and girls is explained by differences among cases of depression rather than the scores of non-depressed. A similar pattern is seen with mean RCADS scores but there are no differences in mean scores between boys and girls among non-depressed.

**Table 6:** BDI-II and RCADS mean scores (SD) by sex and diagnosis

	**DEPRESSED**	**NON-DEPRESSED**	**Differences**
**BDI-II**	***Mean (SD)***	***Mean (SD)***	**Mean (99% CI)**
	***n***	***n***	**p values**
**Boys**	19.09 (8.15)	12.61 (7.47)	6.48 (3.92 – 9.04)
	97	167	<0.001
**Girls**	26.67 (10.15)	14.93 (7.57)	11.74 (9.07 – 14.41)
	204	103	<0.001
Differences	Mean (99% CI)	Mean (99% CI)	
	p values	p values	
	**7.58 (4.76 – 10.41)**	**2.32 (−0.12 – 4.75)**	
	**<0.001**	**0.014**	
RCADS	*Mean (SD)*	*Mean (SD)*	**Mean (99% CI)**
	*n*	*n*	**p values**
**Boys**	22.15 (7.54)	17.64 (7.52)	4.51 (2.00 – 7.02)
	96	163	<0.001
**Girls**	26.81 (8.06)	18.92 (6.75)	7.89 (5.62 – 10.16)
	204	102	<0.001
Differences	Mean (99% CI)	Mean (99% CI)	
	p values	p values	
	**4.66 (2.13 – 7.19)**	**1.28 (−1.09 – 3.65)**	
	**<0.001**	**0.162**	

SD = Standar Deviation; 99% CI = 99% Confidence Interval; n = sample size.

### Criterion validity

**Figure 1:**
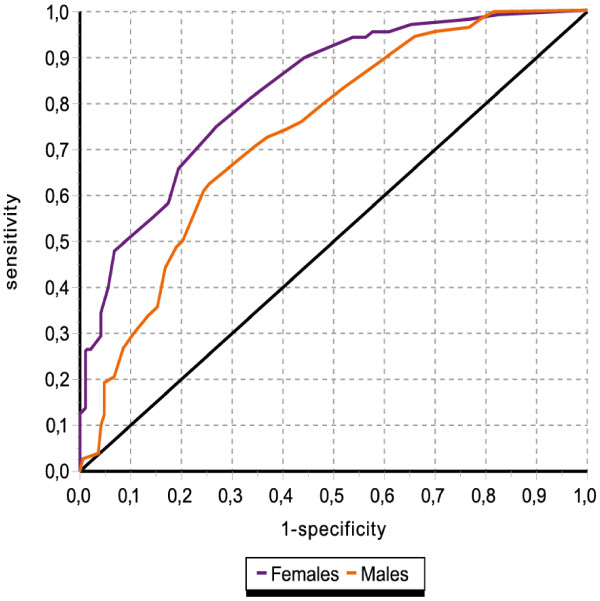
Receiver Operationg Characteristic (ROC curve).

Figure 1 shows the discriminating ability of the BDI-II against a criterion (MINI-KIDS) using ROC curves. The area under the curve for the total score reached a value of 0.81 [95% CI 0.78-0.85; p<0.001] for the total sample. The area under the curve for girls was 0.83 [95% CI 0.78-0.88; p<0.001] whilst it was 0.74 [95% CI 0.68-0.79; p<0.001] for boys, a significant difference according to sex (p=0.022).

Table 7 shows the discriminating ability and precision of the questionnaire for several cut-off points of the total score for either sex separately and for the total sample. We have only displayed validity coefficients for those cut-off points that seemed to be closest to optimal but all other coefficients are available from the authors. Overall the best cut-off point for the whole sample seems to be reached at 16/17 (≥ 17 represents a case) with a sensitivity of 78.7% and a specificity of 69.6%. However optimal cut-off points seem to differ for both boys and girls. In the latter case, a cut-off point at 19/20 offers a better balance in validity coefficients (sensitivity 74.5% and specificity 73.8%) whilst a cut-off point of 13/14 offers a reasonable trade-off between sensitivity (72.2%) and specificity (64.1%) for boys.

**Table 7:** Criterion validity coefficients of BDI-II according to MINI-KIDS

	Cut-off point 13/14*			**Cut-off point 16/17**			**Cut-off point 19/20**		
	**% (95% CI)**			**% (95% CI)**			**% (95% CI)**		
**Index**	**Total**	**Boys**	**Girls**	**Total**	**Boys**	**Girls**	**Total**	**Boys**	**Girls**
**SEN**^**a**^	87.4	72.2	94.6	78.7	55.7	89.7	64.8	44.3	74.5
	(83.5-91.3)	(62.7-81.6)	(91.3-97.9)	(73.9-83.5)	(45.3-66.1)	(85.3-94.1)	(59.2-70.3)	(33.9-54.7)	(68.3-80.7)
**SPE**^**b**^	56.3	64.1	43.7	69.6	77.8	56.3	79.6	83.2	73.8
	(50.2-62.4)	(56.5-71.6)	(33.6-53.7)	(64.0-75.3)	(71.2-84.4)	(46.2-66.4)	(74.6.6-84.6)	(77.3-89.2)	(64.8-82.8)
**PPV**^**c**^	69.0	53.8	76.9	74.3	59.3	80.3	78.0	60.6	84.9
	(64.2-73.8)	(44.9-62.8)	(71.5-82.3)	(69.3-79.2)	(48.7-70.0)	(74.9-85.6)	(72.7-83.3)	(48.5-72.6)	(79.4-90.4)
**NPV**^**d**^	80.0	79.8	80.4	74.6	75.1	73.4	67.9	72.0	59.4
	(74.0-85.9)	(72.7-87.0)	(69.1-91.7)	(69.0-80.2)	(68.4-81.9)	(63.0-83.8)	(61.7-72.3)	(65.4-78.6)	(50.5-68.3)
**YI**^**e**^	0.44	0.36	0.38	0.48	0.34	0.46	0.44	0.28	0.48
	(0.37-0.51)	(0.25-0.48)	(0.28-0.48)	(0.41-0.56)	(0.22-0.45)	(0.36-0.56)	(0.38-0.52)	(0.16-0.39)	(0.38-0.59)

^*^≥14 represents a case; ^a^ = Sensitivity; ^b^ = Specificity; ^c^ = Positive predictive value; ^d^ = Negative predictive value; ^e^ = Youden Index.

## Discussion

As far as we are aware this is the first criterion validity study of the Beck Depression Inventory (BDI-II) among adolescents in Latin America. Overall the questionnaire had good psychometric properties with good internal consistency and good capacity to discriminate between cases and non-cases of depression. We think that a single general factor represents the best factorial solution for this questionnaire with this population. We found that the optimal cut-off point differed according to sex, with the optimal cut-off points being much higher for girls than boys. This is an interesting finding because most of the time cut-off points are established for total samples without considering differences across sexes and/or other attributes, something that may result in significant misclassification. These sex discrepancies were primarily explained by differences in scores among those with depression rather than among those without depression.

The main strength of this study is that we tested criterion validity using a standard psychiatric interview administered independently to ascertain caseness. Interviewers were blind to the results of the questionnaires and the interview was conducted less than 72 hours after the administration of the questionnaire. One of the reasons to explain the absence of criterion validity studies in this field is because of the practical problems as well as resources needed to carry out psychiatric interviews. There are also some limitations. Our sample was of moderate size and stratified according to results to the questionnaire (BDI-II). The sample was also restricted to students from lower socio-economic status and within a limited age range. Finally we were unable to vary the order of administration of the measures for practical reasons.

One of the most salient findings of this study is the clear difference in BDI-II total scores between boys and girls. The origin of these sex differences can only be speculated and it certainly deserves more research. Most evidence suggests that there are true differences in the prevalence of depression according to sex [73-76]. Previous reports had suggested that it may be important to consider why male and female adolescents show different symptom profiles [33,76]. For instance, adolescent girls may be more willing to recognize emotional feelings or they may truly experience more emotional symptoms. In our study girls scored much higher than boys in both the depression and anxiety scales. However we found these sex differences mostly among clinically depressed adolescents and not among non-depressed individuals suggesting that it is only when adolescents are clinically depressed that these sex differences in symptoms reported become important. One could imply that depression might have a different impact in boys and girls so that the latter would report more symptoms but it is also possible that a non-depressed population will also have fewer symptoms and this will attenuate any potential differences across sexes. Regardless of the reasons to explain these differences the fact remains that if the same cut-off point is used across sexes, misclassification is likely. In the end the decision of which cut-off point to choose will depend on what is more important, improving the capacity to detect cases or identify normal individuals.

Our overall proposed cut-off point of 16/17 is higher than that suggested in previous studies with diverse populations [26,28,77]. The discriminant capacity of the questionnaire, represented by the area under the ROC curve, was excellent, being better in girls than boys. If we had not estimated cut-off points independently for each sex we would be advising the use this overall cut-off point with this population. However the analysis by sex revealed that there were substantial differences in optimal cut-off points across sexes. If we had used a cut-off point of 16/17 for both boys and girls, the positive predictive value of the questionnaire among boys would be 59.3% and among girls 80.3%. In other words of all the cases detected by the instrument among boys only 59.3% would be true cases according to the interview (gold standard) whereas in girls 80.3% of those detected by the instrument would be true cases. The capacity to predict cases in boys and girls vary substantially depending on the cut-off point even in high prevalence situations, such as in this study. In previous papers we had identified similar issues related to the socio-economic or cultural status of respondents [44,45].

The BDI-II showed good psychometric qualities. Reliability and internal consistency was high, in keeping with other studies [32,34,36,38] and items were highly correlated. Each item seem to be measuring the same latent variable, but with possibly different degree of precision and different amount of error. Based on the analysis of invariance it seems reasonable to conclude that the same construct seems to apply to both boys and girls. However girls seem to have larger standard errors, most notable for the items ‘crying’ and ‘loss of libido’. Responses to both items are probably influenced by social desirability norms, which may differ between boys and girls. Other studies in adolescents have also encountered similar issues [31,33,78], suggesting that certain items may behave differently with different populations. A study that asked ‘experts’ to rate the relevance of BDI-II items for diagnosing depression among adolescents and asked adolescents themselves about the best questions to report their feelings found that ‘loss of libido’ was the least useful item [31]. Unsurprisingly given the age of these individuals, the ‘loss of libido’ item achieved the lowest mean among all items in both sexes. These findings should inform other researchers about the importance of considering the meaning of items and social norms that may influence responses. Certain questions may be more appropriate for inclusion in studies with adult rather than young populations. Besides this the message that emerges over and over again is that of the need to validate instruments with the populations were they are intended to be used.

The EFA by parallel analysis showed a clear one factor solution, although the proportion of the variance explained by this factor can only be regarded as moderate. This one factor solution was supported by the CFA according to sex. Several other studies have looked at the factor structure of this questionnaire but most of them have not used parallel analysis, which is now regarded as the best approach to ascertain the number of factors to derive from scales. These previous studies have suggested different factor structures with some describing three factors or more [33,79], others suggesting a two-factor structure [26,48,49,80], whilst other studies have suggested that a one general factor is the most appropriate solution [32,81]. It is interesting to note that there seems to be marked variability among studies in terms of the specific items that load into different factors. A single general factor is in keeping with the idea of summing all items to generate a total score reflecting severity, as suggested in the manual of the BDI-II and ratified by a panel of experts in another study [31].

## Conclusions

Symptom questionnaires are often used to identify potential cases without any prior validation to determine the best cut-off points. This practice can lead to substantial misclassification. Although the Beck Depression Inventory (BDI-II) has been frequently used among adolescents in Latin America this seems to be the first criterion validity study. The questionnaire seemed to be good discriminating cases from non-cases of depression. The data supports a single general factor as the best factorial solution with this population. There were substantial sex differences in symptom profiles and most importantly in the optimal cut-off points for girls and boys. If the BDI-II is to be used as a binary instrument through established cut-off points we recommend that these are calculated independently for both sexes. Studies using questionnaires with the same cut-off points for boys and girls may be providing inaccurate estimates and misleading support to the existence of sex differences in depression. Although it is essential that brief self-reported questionnaires are validated with the populations that will be used with, this is unfortunately still the exception rather than the rule. Further replication of these results in other settings and cultures would be important to determine if these findings are specific to this setting or applicable to other cultures.

## Competing interests

The authors declare that they have no competing interests.

## Authors’ contributions

RA, AM, and RF conceived the study and led the bid to secure funding for this work. JM-M analysed the data. SB and RF were responsible for the fieldwork. RA and JM-M wrote the first draft. All authors read and approved the final manuscript.

## Pre-publication history

The pre-publication history for this paper can be accessed here:

http://www.biomedcentral.com/1471-244X/13/122/prepub

## Supplementary Material

Additional file 1 Polychoric correlations. Click here for file
