# Supplementary material for: Detecting depression among adolescents in Santiago, Chile: sex differences
Source: BMC Psychiatry. 2013 Apr 23;13:122. doi: 10.1186/1471-244X-13-122 (PMC3637822; doi:10.1186/1471-244X-13-122)
Supplement: Additional file 1 — Polychoric correlations. [file 1471-244X-13-122-S1.doc]

**Additional file 1: Polychoric correlations**

**1.1. Polychoric correlation for the total sample**

| **Variables** |  | **1** | **2** | **3** | **4** | **5** | **6** | **7** | **8** | **9** | **10** | **11** | **12** | **13** | **14** | **15** | **16** | **17** | **18** | **19** | **20** |
| --- | --- | --- | --- | --- | --- | --- | --- | --- | --- | --- | --- | --- | --- | --- | --- | --- | --- | --- | --- | --- | --- |
|  |  |  |  |  |  |  |  |  |  |  |  |  |  |  |  |  |  |  |  |  |  |
| Item nº 1 |  |  |  |  |  |  |  |  |  |  |  |  |  |  |  |  |  |  |  |  |  |
| Item nº 2 |  | 0.47 |  |  |  |  |  |  |  |  |  |  |  |  |  |  |  |  |  |  |  |
| Item nº 3 |  | 0.45 | 0.46 |  |  |  |  |  |  |  |  |  |  |  |  |  |  |  |  |  |  |
| Item nº 4 |  | 0.36 | 0.35 | 0.36 |  |  |  |  |  |  |  |  |  |  |  |  |  |  |  |  |  |
| Item nº 5 |  | 0.41 | 0.35 | 0.42 | 0.30 |  |  |  |  |  |  |  |  |  |  |  |  |  |  |  |  |
| Item nº 6 |  | 0.41 | 0.34 | 0.27 | 0.20 | 0.26 |  |  |  |  |  |  |  |  |  |  |  |  |  |  |  |
| Item nº 7 |  | 0.39 | 0.36 | 0.42 | 0.40 | 0.36 | 0.28 |  |  |  |  |  |  |  |  |  |  |  |  |  |  |
| Item nº 8 |  | 0.40 | 0.30 | 0.38 | 0.32 | 0.44 | 0.31 | 0.37 |  |  |  |  |  |  |  |  |  |  |  |  |  |
| Item nº 9 |  | 0.50 | 0.35 | 0.37 | 0.33 | 0.38 | 0.34 | 0.35 | 0.33 |  |  |  |  |  |  |  |  |  |  |  |  |
| Item nº 10 |  | 0.42 | 0.24 | 0.23 | 0.29 | 0.27 | 0.26 | 0.24 | 0.33 | 0.35 |  |  |  |  |  |  |  |  |  |  |  |
| Item nº 11 |  | 0.28 | 0.21 | 0.21 | 0.19 | 0.23 | 0.23 | 0.21 | 0.23 | 0.21 | 0.18 |  |  |  |  |  |  |  |  |  |  |
| Item nº 12 |  | 0.34 | 0.37 | 0.27 | 0.41 | 0.26 | 0.19 | 0.38 | 0.28 | 0.29 | 0.24 | 0.22 |  |  |  |  |  |  |  |  |  |
| Item nº 13 |  | 0.30 | 0.30 | 0.30 | 0.35 | 0.35 | 0.22 | 0.33 | 0.30 | 0.25 | 0.25 | 0.24 | 0.37 |  |  |  |  |  |  |  |  |
| Item nº 14 |  | 0.46 | 0.44 | 0.49 | 0.34 | 0.48 | 0.32 | 0.47 | 0.43 | 0.46 | 0.32 | 0.24 | 0.40 | 0.33 |  |  |  |  |  |  |  |
| Item nº 15 |  | 0.42 | 0.32 | 0.35 | 0.38 | 0.29 | 0.21 | 0.35 | 0.32 | 0.37 | 0.28 | 0.16 | 0.41 | 0.36 | 0.40 |  |  |  |  |  |  |
| Item nº 16 |  | 0.19 | 0.14 | 0.20 | 0.16 | 0.24 | 0.10 | 0.14 | 0.21 | 0.19 | 0.18 | 0.17 | 0.22 | 0.15 | 0.20 | 0.26 |  |  |  |  |  |
| Item nº 17 |  | 0.39 | 0.27 | 0.29 | 0.32 | 0.31 | 0.24 | 0.34 | 0.36 | 0.35 | 0.32 | 0.28 | 0.36 | 0.24 | 0.41 | 0.35 | 0.19 |  |  |  |  |
| Item nº 18 |  | 0.21 | 0.20 | 0.20 | 0.17 | 0.24 | 0.11 | 0.25 | 0.25 | 0.22 | 0.22 | 0.17 | 0.27 | 0.22 | 0.28 | 0.26 | 0.23 | 0.21 |  |  |  |
| Item nº 19 |  | 0.29 | 0.27 | 0.25 | 0.21 | 0.23 | 0.20 | 0.25 | 0.22 | 0.24 | 0.28 | 0.28 | 0.30 | 0.23 | 0.26 | 0.29 | 0.23 | 0.22 | 0.27 |  |  |
| Item nº 20 |  | 0.41 | 0.35 | 0.39 | 0.38 | 0.33 | 0.25 | 0.30 | 0.41 | 0.37 | 0.28 | 0.27 | 0.40 | 0.31 | 0.38 | 0.46 | 0.29 | 0.37 | 0.34 | 0.35 |  |
| Item nº 21 |  | 0.38 | 0.32 | 0.25 | 0.26 | 0.22 | 0.25 | 0.24 | 0.25 | 0.31 | 0.26 | 0.21 | 0.34 | 0.23 | 0.30 | 0.30 | 0.10 | 0.23 | 0.27 | 0.15 | 0.31 |

Determinant < 0.01; KMO = 0.94; Bartlett's statistic = 3,672.30 (df = 210) p < 0.001.

**1.2. Polychoric correlation for the boys sample**

| **Variables** |  | **1** | **2** | **3** | **4** | **5** | **6** | **7** | **8** | **9** | **10** | **11** | **12** | **13** | **14** | **15** | **16** | **17** | **18** | **19** | **20** |
| --- | --- | --- | --- | --- | --- | --- | --- | --- | --- | --- | --- | --- | --- | --- | --- | --- | --- | --- | --- | --- | --- |
|  |  |  |  |  |  |  |  |  |  |  |  |  |  |  |  |  |  |  |  |  |  |
| Item nº 1 |  |  |  |  |  |  |  |  |  |  |  |  |  |  |  |  |  |  |  |  |  |
| Item nº 2 |  | 0.56 |  |  |  |  |  |  |  |  |  |  |  |  |  |  |  |  |  |  |  |
| Item nº 3 |  | 0.51 | 0.52 |  |  |  |  |  |  |  |  |  |  |  |  |  |  |  |  |  |  |
| Item nº 4 |  | 0.40 | 0.41 | 0.35 |  |  |  |  |  |  |  |  |  |  |  |  |  |  |  |  |  |
| Item nº 5 |  | 0.36 | 0.36 | 0.47 | 0.34 |  |  |  |  |  |  |  |  |  |  |  |  |  |  |  |  |
| Item nº 6 |  | 0.38 | 0.33 | 0.27 | 0.18 | 0.21 |  |  |  |  |  |  |  |  |  |  |  |  |  |  |  |
| Item nº 7 |  | 0.41 | 0.39 | 0.47 | 0.42 | 0.37 | 0.28 |  |  |  |  |  |  |  |  |  |  |  |  |  |  |
| Item nº 8 |  | 0.34 | 0.31 | 0.38 | 0.27 | 0.38 | 0.24 | 0.37 |  |  |  |  |  |  |  |  |  |  |  |  |  |
| Item nº 9 |  | 0.51 | 0.39 | 0.34 | 0.35 | 0.29 | 0.39 | 0.43 | 0.31 |  |  |  |  |  |  |  |  |  |  |  |  |
| Item nº 10 |  | 0.42 | 0.23 | 0.21 | 0.37 | 0.19 | 0.30 | 0.29 | 0.25 | 0.36 |  |  |  |  |  |  |  |  |  |  |  |
| Item nº 11 |  | 0.30 | 0.21 | 0.13 | 0.20 | 0.25 | 0.20 | 0.23 | 0.19 | 0.25 | 0.24 |  |  |  |  |  |  |  |  |  |  |
| Item nº 12 |  | 0.31 | 0.36 | 0.23 | 0.41 | 0.23 | 0.17 | 0.29 | 0.21 | 0.28 | 0.18 | 0.13 |  |  |  |  |  |  |  |  |  |
| Item nº 13 |  | 0.28 | 0.29 | 0.31 | 0.35 | 0.35 | 0.26 | 0.30 | 0.28 | 0.20 | 0.30 | 0.24 | 0.28 |  |  |  |  |  |  |  |  |
| Item nº 14 |  | 0.47 | 0.49 | 0.50 | 0.32 | 0.44 | 0.31 | 0.43 | 0.40 | 0.43 | 0.35 | 0.18 | 0.38 | 0.29 |  |  |  |  |  |  |  |
| Item nº 15 |  | 0.41 | 0.37 | 0.30 | 0.37 | 0.25 | 0.22 | 0.31 | 0.17 | 0.37 | 0.27 | 0.15 | 0.34 | 0.35 | 0.34 |  |  |  |  |  |  |
| Item nº 16 |  | 0.18 | 0.20 | 0.19 | 0.10 | 0.24 | 0.07 | 0.11 | 0.15 | 0.10 | 0.09 | 0.19 | 0.15 | 0.13 | 0.14 | 0.22 |  |  |  |  |  |
| Item nº 17 |  | 0.27 | 0.27 | 0.17 | 0.29 | 0.25 | 0.23 | 0.27 | 0.29 | 0.36 | 0.31 | 0.19 | 0.25 | 0.18 | 0.34 | 0.22 | 0.10 |  |  |  |  |
| Item nº 18 |  | 0.18 | 0.16 | 0.17 | 0.10 | 0.21 | 0.01 | 0.20 | 0.12 | 0.18 | 0.16 | 0.15 | 0.20 | 0.15 | 0.22 | 0.17 | 0.21 | 0.13 |  |  |  |
| Item nº 19 |  | 0.30 | 0.21 | 0.26 | 0.19 | 0.23 | 0.16 | 0.25 | 0.16 | 0.22 | 0.20 | 0.29 | 0.20 | 0.19 | 0.19 | 0.27 | 0.22 | 0.15 | 0.18 |  |  |
| Item nº 20 |  | 0.41 | 0.40 | 0.38 | 0.34 | 0.34 | 0.23 | 0.29 | 0.36 | 0.37 | 0.23 | 0.26 | 0.35 | 0.32 | 0.36 | 0.42 | 0.25 | 0.28 | 0.29 | 0.36 |  |
| Item nº 21 |  | 0.45 | 0.37 | 0.24 | 0.25 | 0.18 | 0.31 | 0.27 | 0.23 | 0.42 | 0.39 | 0.23 | 0.31 | 0.23 | 0.40 | 0.30 | 0.03 | 0.22 | 0.20 | 0.14 | 0.34 |

Determinant < 0.01; KMO = 0.91; Bartlett's statistic = 1,714.20 (df = 210) p < 0.001.

**1.3. Polychoric correlation for the girls sample**

| **Variables** |  | **1** | **2** | **3** | **4** | **5** | **6** | **7** | **8** | **9** | **10** | **11** | **12** | **13** | **14** | **15** | **16** | **17** | **18** | **19** | **20** |
| --- | --- | --- | --- | --- | --- | --- | --- | --- | --- | --- | --- | --- | --- | --- | --- | --- | --- | --- | --- | --- | --- |
|  |  |  |  |  |  |  |  |  |  |  |  |  |  |  |  |  |  |  |  |  |  |
| Item nº 1 |  |  |  |  |  |  |  |  |  |  |  |  |  |  |  |  |  |  |  |  |  |
| Item nº 2 |  | 0.34 |  |  |  |  |  |  |  |  |  |  |  |  |  |  |  |  |  |  |  |
| Item nº 3 |  | 0.34 | 0.40 |  |  |  |  |  |  |  |  |  |  |  |  |  |  |  |  |  |  |
| Item nº 4 |  | 0.27 | 0.29 | 0.33 |  |  |  |  |  |  |  |  |  |  |  |  |  |  |  |  |  |
| Item nº 5 |  | 0.38 | 0.33 | 0.36 | 0.24 |  |  |  |  |  |  |  |  |  |  |  |  |  |  |  |  |
| Item nº 6 |  | 0.37 | 0.34 | 0.26 | 0.20 | 0.28 |  |  |  |  |  |  |  |  |  |  |  |  |  |  |  |
| Item nº 7 |  | 0.33 | 0.31 | 0.34 | 0.35 | 0.31 | 0.28 |  |  |  |  |  |  |  |  |  |  |  |  |  |  |
| Item nº 8 |  | 0.39 | 0.26 | 0.33 | 0.31 | 0.44 | 0.24 | 0.32 |  |  |  |  |  |  |  |  |  |  |  |  |  |
| Item nº 9 |  | 0.45 | 0.30 | 0.35 | 0.28 | 0.40 | 0.39 | 0.23 | 0.25 |  |  |  |  |  |  |  |  |  |  |  |  |
| Item nº 10 |  | 0.30 | 0.23 | 0.15 | 0.17 | 0.23 | 0.30 | 0.10 | 0.23 | 0.24 |  |  |  |  |  |  |  |  |  |  |  |
| Item nº 11 |  | 0.26 | 0.20 | 0.27 | 0.19 | 0.22 | 0.20 | 0.19 | 0.27 | 0.18 | 0.14 |  |  |  |  |  |  |  |  |  |  |
| Item nº 12 |  | 0.31 | 0.39 | 0.27 | 0.37 | 0.26 | 0.17 | 0.39 | 0.27 | 0.29 | 0.19 | 0.27 |  |  |  |  |  |  |  |  |  |
| Item nº 13 |  | 0.21 | 0.28 | 0.26 | 0.33 | 0.30 | 0.26 | 0.31 | 0.25 | 0.23 | 0.12 | 0.24 | 0.36 |  |  |  |  |  |  |  |  |
| Item nº 14 |  | 0.39 | 0.38 | 0.44 | 0.31 | 0.48 | 0.31 | 0.46 | 0.39 | 0.44 | 0.21 | 0.27 | 0.36 | 0.31 |  |  |  |  |  |  |  |
| Item nº 15 |  | 0.35 | 0.26 | 0.33 | 0.36 | 0.27 | 0.22 | 0.32 | 0.31 | 0.30 | 0.11 | 0.16 | 0.41 | 0.32 | 0.39 |  |  |  |  |  |  |
| Item nº 16 |  | 0.20 | 0.08 | 0.19 | 0.19 | 0.22 | 0.07 | 0.13 | 0.21 | 0.23 | 0.20 | 0.15 | 0.28 | 0.14 | 0.21 | 0.26 |  |  |  |  |  |
| Item nº 17 |  | 0.41 | 0.25 | 0.32 | 0.30 | 0.31 | 0.23 | 0.34 | 0.33 | 0.29 | 0.23 | 0.34 | 0.36 | 0.23 | 0.41 | 0.38 | 0.22 |  |  |  |  |
| Item nº 18 |  | 0.17 | 0.21 | 0.18 | 0.19 | 0.23 | 0.01 | 0.24 | 0.29 | 0.19 | 0.18 | 0.17 | 0.30 | 0.23 | 0.28 | 0.27 | 0.22 | 0.23 |  |  |  |
| Item nº 19 |  | 0.23 | 0.30 | 0.19 | 0.19 | 0.19 | 0.16 | 0.21 | 0.19 | 0.20 | 0.25 | 0.27 | 0.32 | 0.21 | 0.27 | 0.24 | 0.22 | 0.21 | 0.31 |  |  |
| Item nº 20 |  | 0.37 | 0.29 | 0.36 | 0.38 | 0.28 | 0.23 | 0.25 | 0.38 | 0.31 | 0.22 | 0.27 | 0.40 | 0.26 | 0.35 | 0.44 | 0.29 | 0.39 | 0.34 | 0.29 |  |
| Item nº 21 |  | 0.28 | 0.27 | 0.22 | 0.25 | 0.22 | 0.31 | 0.19 | 0.22 | 0.20 | 0.13 | 0.20 | 0.33 | 0.20 | 0.21 | 0.25 | 0.12 | 0.19 | 0.29 | 0.12 | 0.26 |

Determinant < 0.01; KMO = 0.92; Bartlett's statistic = 1,767.20 (df = 210) p < 0.001.
